# Analysis of gene order data supports vertical inheritance of the leukotoxin operon and genome rearrangements in the 5' flanking region in genus *Mannheimia*

**DOI:** 10.1186/1471-2148-7-184

**Published:** 2007-10-03

**Authors:** Jesper Larsen, Peter Kuhnert, Joachim Frey, Henrik Christensen, Magne Bisgaard, John E Olsen

**Affiliations:** 1 Department of Veterinary Pathobiology, Faculty of Life Sciences, University of Copenhagen, Stigbøjlen 4, DK-1870 Frederiksberg C, Denmark; 2 Institute of Veterinary Bacteriology, University of Berne, Länggass-Strasse 122, CH-3012 Berne, Switzerland

## Abstract

**Background:**

The *Mannheimia *subclades belong to the same bacterial genus, but have taken divergent paths toward their distinct lifestyles. For example, *M*. *haemolytica *+ *M*. *glucosida *are potential pathogens of the respiratory tract in the mammalian suborder Ruminantia, whereas *M*. *ruminalis*, the supposed sister group, lives as a commensal in the ovine rumen. We have tested the hypothesis that vertical inheritance of the leukotoxin (*lktCABD*) operon has occurred from the last common ancestor of genus *Mannheimia *to any ancestor of the diverging subclades by exploring gene order data.

**Results:**

We examined the gene order in the 5' flanking region of the leukotoxin operon and found that the 5' flanking gene strings, *hslVU*-*lapB*-*artJ*-*lktC *and *xylAB*-*lktC*, are peculiar to *M*. *haemolytica *+ *M*. *glucosida *and *M*. *granulomatis*, respectively, whereas the gene string *hslVU*-*lapB*-*lktC *is present in *M*. *ruminalis*, the supposed sister group of *M*. *haemolytica *+ *M*. *glucosida*, and in the most ancient subclade *M*. *varigena*. In *M*. *granulomatis*, we found remnants of the gene string *hslVU*-*lapB*-*lktC *in the *xylB*-*lktC *intergenic region.

**Conclusion:**

These observations indicate that the gene string *hslVU*-*lapB*-*lktC *is more ancient than the *hslVU*-*lapB*-*artJ*-*lktC *and *xylAB*-*lktC *gene strings. The presence of (remnants of) the ancient gene string *hslVU*-*lapB*-*lktC *among any subclades within genus *Mannheimia *supports that it has been vertically inherited from the last common ancestor of genus *Mannheimia *to any ancestor of the diverging subclades, thus reaffirming the hypothesis of vertical inheritance of the leukotoxin operon. The presence of individual 5' flanking regions in *M*. *haemolytica *+ *M*. *glucosida *and *M*. *granulomatis *reflects later genome rearrangements within each subclade. The evolution of the novel 5' flanking region in *M*. *haemolytica *+ *M*. *glucosida *resulted in transcriptional coupling between the divergently arranged *artJ *and *lkt *promoters. We propose that the chimeric promoter have led to high level expression of the leukotoxin operon which could explain the increased potential of certain *M*. *haemolytica *+ *M*. *glucosida *strains to cause a particular type of infection.

## Background

Genomes are evolutionary unstable structures that are subject to frequent dynamic rearrangements [1-4]. Huynen and Bork [5] showed that overall gene order and evolutionary distance are inversely correlated until a saturation level is reached. In a more recent analysis [4] 25 genomes were screened looking for conservation of gene order. This analysis suggested that the saturation level is dictated by functional constraints on operon structure and that only 5–25% of the genes in bacterial and archaeal genomes belong to gene strings that are conserved in at least two genomes. However, some phylogenetic lineages show remarkable conservation and others extensive genome rearrangements [6,7]. There are four types of changes that are thought to affect the order of the genes on the bacterial genome: (i) inversions and translocations are frequently detected when the genomes of even closely related species are compared [8]; (ii) genes may be lost in a single event [9] or as a consequence of multistep deletion of pseudogenes [10,11]; (iii) incorporation of nonhomologous genes or operons throughout the genome by illegitimate recombination in a process known as horizontal gene transfer (HGT) is thought to be a major force in the evolution of gene content of bacterial species [12,13]; (iv) partial duplications of the genome may produce redundant genome segments [14-17].

The genus *Mannheimia *includes strains previously classified as trehalose-negative [*Pasteurella*] *haemolytica *and is one of the most well-defined and robust clusters within the gamma-proteobacterial family of *Pasteurellaceae *Pohl 1981 [18]. The *Mannheimia *subclades have taken divergent paths toward their distinct lifestyles. For example, *M*. *haemolytica *+ *M*. *glucosida *form one of the most recently diverged subclades within genus *Mannheimia *based on 16S rRNA sequences and both species are potential pathogens in the mammalian suborder Ruminantia, whereas *M*. *ruminalis*, the supposed sister group, lives as a commensal in the ovine rumen [18]. Previous works have revealed that the leukotoxin (LktA) protein of *M*. *haemolytica *plays an important role in the evasion and exploitation of the adaptive immune system during pulmonary infection via interactions with host cells [19-21]. The leukotoxin (*lkt*) operon codes for four proteins: an internal acyltransferase, encoded by *lktC *[22]; the structural toxin, encoded by *lktA*, which belongs to the *Escherichia coli *HlyA-like subfamily of cytotoxic RTX (repeats in toxin) proteins [22]; an inner membrane protein with a cytoplasmic ATP binding cassette (ABC) domain, encoded by *lktB*, which pumps out LktA protein via interaction with the C terminus of LktA [23]; and a membrane fusion protein, encoded by *lktD*, which forms a bridge between the inner and outer membranes [23]. The genes for these four proteins are physically adjacent on the chromosome and are transcribed as *lktCA *or *lktCABD *messages [24,25].

In a recent work, Larsen et al. [26] performed phylogenetic and compositional analyses in order to explore the origin and fate of the *lkt *operon in *M*. *haemolytica *+ *M*. *glucosida*. The scenario derived from this work suggested that it has been vertically inherited from the last common ancestor of genus *Mannheimia *to any ancestor of the diverging subclades, although several strains belonging to *M*. *ruminalis *have lost the operon. However, reconstruction of the *rtxA *tree provided strong support for a sister group relationship between *M*. *haemolytica *+ *M*. *glucosida *and *M*. *ruminalis *on one hand and the more distantly related taxon [*Pasteurella*] *trehalosi *on the other. This incongruence between gene trees and organismal phylogenies could arise from HGT of the *lkt *operon from [*P*.] *trehalosi *to a common ancestor of these *Mannheimia *subclades.

In this article, we have examined the compatibility of the gene order in the 5' flanking region of the *lkt *operon (called hereafter the 5' flanking gene order) in the genomes of representatives from the four major subclades within genus *Mannheimia *with the Larsen and coworkers' [26] hypothesis of vertical inheritance. Under the assumption that closely related species accumulate fewer rearrangements than the distant ones, vertical inheritance should result in *lkt *operons belonging to gene strings that are conserved in all genomes. On the other hand, HGT would result in disruption of the 5' flanking gene order (breakpoints) between the same genomes.

## Results

### Sequencing of 5' flanking regions

The 5' flanking gene string in *M*. *haemolytica *serotype 1 str. PHL101 [GenBank:M59210] contains four genes considered to code for functionally and physically interacting proteins in an L-arginine ABC transporter [27]. In order to examine the compatibility of the 5' flanking gene order with the Larsen and coworkers' [26] hypothesis of vertical inheritance, we here analyzed homologous genome segments in representatives from the four major subclades within genus *Mannheimia*. We retrieved data (contig74) from the annotated genome sequence of *M*. *haemolytica *serotype 1 str. PHL213 [GenBank:NZ_AASA01000031]. For the remaining *Mannheimia *strains, genome segments were first cloned into the Zap Express vector and packaged into the Gigapack III Gold Packaging Extract. The resulting phagemid libraries were then screened for segments of 5' flanking genes by the plaque hybridization method using a 542 bp fragment of the *lktCA *genes as a probe.

**Figure 1:**
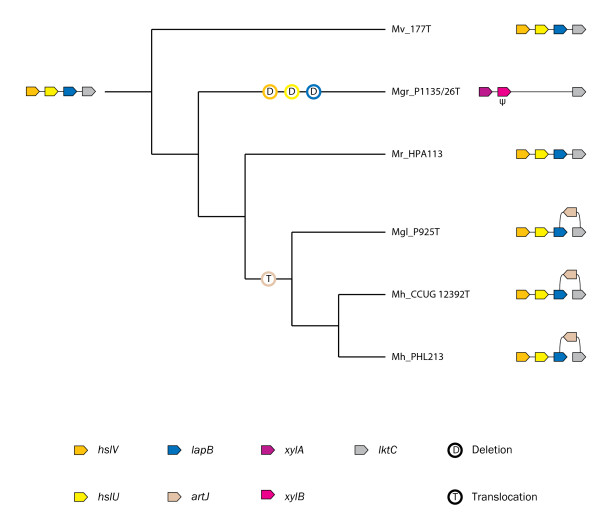
**Evolution of the 5' flanking gene order in the *Mannheimia *genomes analyzed in this study**. The organismal cladogram that describes bifurcation order was adapted from Larsen et al. [26]. Colored arrows indicate orthology as in Figure 2. Gene names are those reported in protein databases or have been assigned by us on the basis of orthology relationships. Pseudogenes are indicated by a "Ψ". The two types of genome rearrangements are indicated by circles. Nomenclature: sequence names contain abbreviations of the taxonomic group (genus and species) followed by the corresponding strain ID as listed in Table 1.

The sizes of the sequenced genome segments varied from 1,383 to 9,126 bp (Table 1). Our initial search for putative orthologs suggested that the 5' flanking gene strings, *hslVU*-*lapB*-*artI*/*artJ*-*lktC *and *xylAB*-*lktC*, are peculiar to *M*. *haemolytica *+ *M*. *glucosida *and *M*. *granulomatis*, respectively, whereas the gene string *hslVU*-*lapB*-*lktC *is present in *M*. *ruminalis*, the supposed sister group of *M*. *haemolytica *+ *M*. *glucosida*, and in the most ancient subclade *M*. *varigena *(Figure 1). It should be noted that for one open reading frame (ORF) we retrieved two closely related proteins, ArtI and ArtJ, in a subset of the genomes and that the corresponding *artI*/*artJ *gene is located on the opposite strand immediately upstream of the *lkt *operon.

**Table 1:** Strains used in this study

Subclade	Taxon^a^	Strain ID	Host	Country	Size of genome segment (bp)	GenBank
	*M*. *haemolytica *(Mh)					
I	Biogroup 1	PHL213	Ruminantia			[GenBank:NZ_AASA01000031]
I	Biogroup 1	CCUG 12392^T^	Ruminantia	UK	1,383	[GenBank:AY425276]
	*M*. *glucosida *(Mgl)					
I	Biogroup 3B	P925^T^	Ruminantia	Scotland	3,373	[GenBank:AY425277]
	*M*. *ruminalis *(Mr)					
II	Bt 18 biovar 2	HPA113	Ruminantia	UK	9,126	[GenBank:AY425280]
	*M*. *granulomatis *(Mgr)					
III	[*P*.] *granulomatis*	P1135/26^T^	Ruminantia	Brazil	5,233	[GenBank:AY425278]
	*M*. *varigena *(Mv)					
IV	Biogroup 6	177^T^	Ruminantia	Germany	4,765	[GenBank:AY425279]

^a ^Bt = Bisgaard taxon.

### Description of genes

Prior to this work, Highlander et al. [27] analyzed the 5' flanking genes in *M*. *haemolytica *serotype 1 str. PHL101 and noted that the gene cluster *lapCABT*, corresponding to the gene string *hslV*-*hslU*-*lapB*-*artI*/*artJ*, codes for functionally and physically interacting proteins in an L-arginine ABC transporter. A close look at the Lap proteins showed that updated annotations were already available in SWISS-PROT (Table 2). The SWISS-PROT entry for the LapT protein [SWISS-PROT:P49618] showed that it is annotated as "arginine-binding periplasmic protein". PSI-BLAST searches of the COG database revealed that LapT belongs to COG0834 (annotated as "ABC-type amino acid transport/signal transduction systems, periplasmic component/domain"), thus supporting the updated annotation in SWISS-PROT (Table 2). Our initial search for putative orthologs retrieved two closely related proteins, ArtI and ArtJ, in a subset of the genomes as explained above. These proteins belong to the polar amino acid transporter (PAAT) family of the periplasmic binding protein clan [28]. Members of this family bind substrates in the vicinity of the inner membrane with high affinity followed by delivery of the liganded form to a membrane bound complex for translocation. These complexes belong to the family of ABC transporters and are composed of two integral membrane domains and two hydrophilic domains that each contains a conserved nucleotide binding domain (NBD). The different domains are composed of separate protein subunits in prokaryotes, whereas they are generally fused into a single polypeptide in eukaryotes. In *E*. *coli*, the membrane bound complex of the L-arginine ABC transporter ArtQMP_2 _consists of two integral membrane protein subunits, ArtQ and ArtM, and two copies of the nucleotide binding protein subunit ArtP [29]. Previous works revealed that ArtJ from *E*. *coli *and LapT from *M*. *haemolytica *serotype 1 str. PHL101 bind L-arginine with a higher affinity compared to other amino acids based on equilibrium titration experiments [29,30]. On the contrary, the binding affinity for L-arginine by ArtI from *E*. *coli *was one order of magnitude lower compared to ArtJ and was almost identical to the binding affinities for the other amino acids by ArtI and ArtJ [29]. These data suggest that LapT and ArtJ, but not ArtI, are orthologs of each other.

**Table 2:** Homology-based functional characterization of the 5' flanking genes

Ortholog	Size (aa)	SWISS-PROT	COG	COG annotation
*hslV*	173	[SWISS-PROT:P49617]	5405	ATP-dependent protease HslVU (ClpYQ), peptidase subunit
*hslU*	440	[SWISS-PROT:P32180]	1220	ATP-dependent protease HslVU (ClpYQ), ATPase subunit
*lapB*	225	[SWISS-PROT:P32181]	2990	Uncharacterized protein conserved in bacteria
*artI*/*artJ*	237	[SWISS-PROT:P49618]	0834	ABC-type amino acid transport/signal transduction systems, periplasmic component/domain
*xylA*	439		2115	Xylose isomerase
*xylB*	484		1070	Sugar (pentulose and hexulose) kinases

The LapB protein [SWISS-PROT:P32181] is annotated as "membrane protein LapB" in SWISS-PROT. PSI-BLAST searches of the COG database showed that LapB belongs to COG2990 (annotated as "uncharacterized protein conserved in bacteria") (Table 2). These data support that the *lapB *gene could code for an integral membrane protein subunit (ArtQ or ArtM) of an L-arginine ABC transporter as predicted by Highlander et al. [27], but other alternatives has to be considered.

The LapC protein [SWISS-PROT:P49617] has been annotated as "ATP-dependent protease HslV", whereas the LapA protein [SWISS-PROT:P32180] has been annotated as "ATP-dependent Hsl protease ATP-binding subunit HslU" in SWISS-PROT. PSI-BLAST searches of the COG database revealed that LapC and LapA belong to COG5405 (annotated as "ATP-dependent protease HslVU (ClpYQ), peptidase subunit") and COG1220 (annotated as "ATP-dependent protease HslVU (ClpYQ), ATPase subunit"), respectively, thus reaffirming the updated annotations in SWISS-PROT (Table 2). The bacterial ATP-dependent protease HslVU is a homolog of the eukaryotic 26S proteasome [31]. The proteolytic subunit HslV (ClpQ) is a member of the MEROPS peptidase subfamily T01B [32]. Structural information revealed that the HslV protein from *E*. *coli *and the β-subunits of the proteasome catalytic core (20S particle) share ~20% similarity over ~200 amino acid residues and a conserved fold [33]. HslU (ClpY) belongs to the Clp/Hsp100 proteins of the AAA+ superfamily which comprises ClpA, ClpX, and HslU on one hand and ClpB on the other [34]. ClpA, ClpX, and HslU interact with cellular peptidases to form ATP-dependent proteases. The role of these Clp proteins is the unfolding of substrates by energy-dependent threading prior to the delivery to the proteolytic subunit. On the contrary, ClpB from *E*. *coli *and *Thermus thermophilus *and the homolog Hsp104 from *Saccharomyces cerevisiae *do not appear to bind to cellular proteases. Instead they interact with the DnaK/Hsp70 chaperone system to assist in the disaggregation and reactivation of strongly aggregated proteins [35-39]. Clp proteins can be further classified according to the number of NBDs which contain the ATP binding Walker A and B motifs. Class 1 proteins ClpA and ClpB contain two NBDs, whereas class 2 proteins HslU and ClpX contain only one NBD [40,41]. Sequence and structural information has shed light on the domain architecture of the class 2 protein HslU from *E*. *coli *[34,42]. This protein is folded into three distinct domains with the N domain comprising residues 2–109 and 244–332 and the C domain comprising residues 333–443. The intermediate domain (I domain) that is not found in other AAA+ proteins emerges from the N domain at residue 110 and returns to it at residue 243. Multiple alignment of Clp proteins from *E*. *coli *showed that the spacing between the Walker A and B motifs in ClpX and ClpA is ~55 residues, whereas it is 186 residues in HslU due to their separation by the I domain [43]. PSI-BLAST searches of the PDB database and the corresponding multiple alignments showed that the domain architecture of LapA and HslU is conserved, including the spacing between the Walker A and B motifs (184 and 186 residues, respectively) (data not shown). Highlander et al. [27] used homology methods to compare the LapA protein with the Clp/Hsp100 protein ClpA (class 1 protein containing two NBDs), but not HslU (class 2 protein containing only one NBD), and nucleotide binding protein subunits of the ABC transporter family (each containing one NBD). These authors classified LapA as the nucleotide binding protein subunit of an L-arginine ABC transporter (ArtP) based on the spacing between the Walker A and B motifs and the number of NBDs. However, the spacing between these motifs in the closely related nucleotide binding protein subunits ArtP and HisP is only 118 and 126 residues, respectively [29,44], whereas it is 184 residues in LapA and 186 residues in HslU as explained above. These data suggest that LapA and HslU are putative orthologs of each other, thus rejecting the Highlander and coworkers' [27] hypothesis that the *lapA *gene codes for a nucleotide binding protein subunit (ArtP) of an L-arginine ABC transporter.

PSI-BLAST searches of the COG database showed that the XylA and XylB proteins belong to COG2115 (annotated as "xylose isomerase") and COG1070 (annotated as "sugar (pentulose and hexulose) kinases"), respectively (Table 2). Two previous works have revealed that these proteins are involved in the dissimilation of D-xylose through the pentose phosphate pathway in *E*. *coli *and *Salmonella typhimurium *[45,46]. The sugar is first isomerized into D-xylulose by xylose isomerase (XylA), which is a member of the xylose isomerase-like TIM barrel family, and then phosphorylated by xylulokinase (XylB), which belongs to the FGGY family of carbohydrate kinases, to produce D-xylulose 5-phosphate.

### Description of pseudogenes and remnant DNA

The *xylB *gene in *M*. *granulomatis *str. P1135/26^T ^is predicted to code for a short peptide that corresponds to ~57% of the XylB protein. The premature stop codon is located at codon 276 and is the result of frameshift due to a 1 bp insertion at the first position of codon 270 (data not shown). The designation of *xylB *as a pseudogene is compatible with the results of Angen et al. [47], who showed that a single lineage of *M*. *granulomatis*, including strain P1135/26^T^, has lost the ability to utilize D-xylose as a carbon source.

Interestingly, the *lapB *gene in *M*. *haemolytica *serotype 1 str. PHL101 is predicted to code for a short peptide that corresponds to the first ~75% of the LapB protein (data not shown). Multiple alignment revealed that the pseudogene originates from a 1 bp deletion at the second position of codon 217 leading to frameshift and a premature stop codon at codon 225 (data not shown).

If we assume that genes have been inactivated continuously since the divergence of *M*. *granulomatis*, it is expected that some genes have been degraded to such an extent that they are no longer recognizable as pseudogenes. In order to explore the fate of the genes identified in the other *Mannheimia *genomes in the previous step, we performed BLAST searches of the *xylB*-*lktC *intergenic region against 5' flanking gene strings in these genomes. Interestingly, we found a weak but significant hit to the *hslVU *operon in *M*. *haemolytica *serotype 1 str. PHL213 (E-value = 5E-16) due to a 50 bp inverted sequence (data not shown).

### Description of conserved gene strings

Based on the notion that statistically significant conserved gene strings can be confidently predicted to form operons [4], we have analyzed a set of 17 proteobacterial genomes in order to determine the operon organization of the 5' flanking genes. The data set included a balanced number of representatives from the gamma-proteobacterial group, including three *Pasteurellaceae *genomes which are closely related to the *Mannheimia *genomes, and the beta-proteobacterial sister group (Table 3).

**Table 3:** Proteobacterial genomes analyzed in this study

Organism	Class	Order	Family	GenBank
*Bordetella bronchiseptica *RB50	Beta	Burkholderiales	*Alcaligenaceae*	[GenBank:BX470250]
*Ralstonia solanacearum *GM1000	Beta	Burkholderiales	*Burkholderiaceae*	[GenBank:AL646052]
*Neisseria meningitidis *serogroup A strain Z2491	Beta	Neisseriales	*Neisseriaceae*	[GenBank:AL157959]
*Nitrosomonas europaea *ATCC 19718	Beta	Nitrosomonadales	*Nitrosomonadaceae*	[GenBank:AL954747]
*Shewanella oneidensis *MR-1	Gamma	Alteromonadales	*Shewanellaceae*	[GenBank:AE014299]
*Buchnera aphidicola *str. APS	Gamma	Enterobacteriales	*Enterobacteriaceae*	[GenBank:BA000003]
*Escherichia coli *O157:H7 str. Sakai	Gamma	Enterobacteriales	*Enterobacteriaceae*	[GenBank:BA000007]
*Photorhabdus luminescens *subsp. *laumondii *TTO1	Gamma	Enterobacteriales	*Enterobacteriaceae*	[GenBank:BX470251]
*Salmonella enterica *serovar Typhi strain Ty2	Gamma	Enterobacteriales	*Enterobacteriaceae*	[GenBank:AE014613]
*Coxiella burnetii *RSA 493	Gamma	Legionellales	*Coxiellaceae*	[GenBank:AE016828]
*Haemophilus ducreyi *35000 HP	Gamma	Pasteurellales	*Pasteurellaceae*	[GenBank:AE017143]
*Haemophilus influenzae *Rd KW20	Gamma	Pasteurellales	*Pasteurellaceae*	[GenBank:L42023]
*Pasteurella multocida *PM70	Gamma	Pasteurellales	*Pasteurellaceae*	[GenBank:AE004439]
*Pseudomonas aeruginosa *PAO1	Gamma	Pseudomonadales	*Pseudomonadaceae*	[GenBank:AE004091]
*Vibrio vulnificus *YJ016	Gamma	Vibrionales	*Vibrionaceae*	[GenBank:BA000037] [GenBank:BA000038]
*Xanthomonas campestris *pv. *campestris *str. ATCC 33913	Gamma	Xanthomonadales	*Xanthomonadaceae*	[GenBank:AE008922]
*Xylella fastidiosa *9a5c	Gamma	Xanthomonadales	*Xanthomonadaceae*	[GenBank:AE003849]

**Figure 2:**
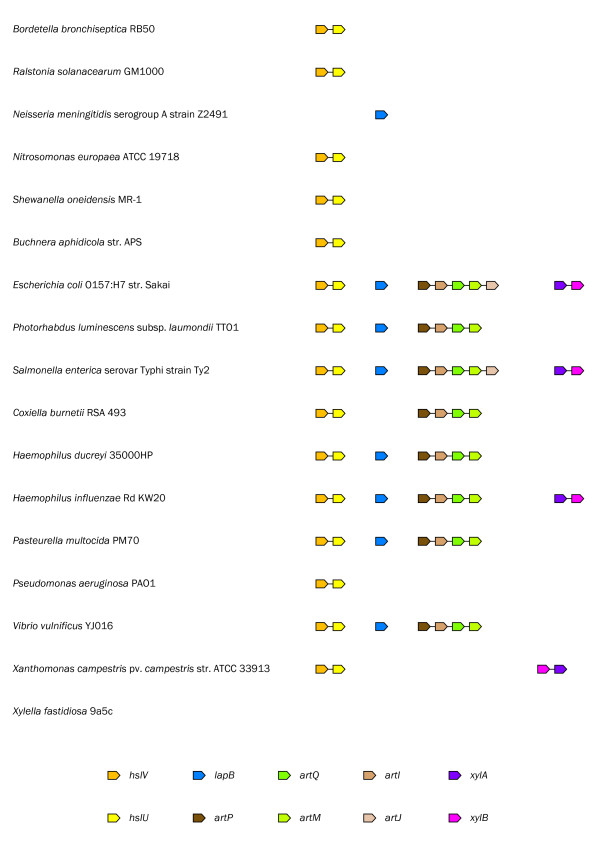
**Conserved gene strings in 17 prokaryotic genomes analyzed in this study**. For each protein searches were performed at the STRING server [57] with a high confidence score > 0.7. Colored arrows indicate orthology. Gene names are those reported in protein databases.

The *hslV *and *hslU *genes from *E*. *coli *are transcribed from a single promoter containing recognition signals for the heat shock transcription factor σ^32^, with consensus sequence STTGAA-N_11–12_-GNCCCCATWT, suggesting that the genes belong to the σ^32 ^regulon [42]. STRING searches revealed that the *hslV *and *hslU *genes are present in 15 genomes, including those of *Pasteurellaceae*, where they belong to the conserved gene string *hslV*-*hslU*, suggesting that these genes comprise an operon (Figure 2). We have shown that the *hslV *and *hslU *genes from *M*. *haemolytica *serotype 1 str. PHL213 are transcribed in the same direction (Figure 1) and that putative -10 and -35 motifs for σ^32 ^are present upstream of *hslV *(data not shown). These results are compatible with the predictions of Chuang et al. [42] and provide clear and robust support for functional and physical interactions between the *hslV *and *hslU *genes.

The *lapB *gene is present in eight of the genomes, including those of *Pasteurellaceae*, but does not belong to any gene string spanning two or more genes, suggesting that the gene comprises its own operon (Figure 2). The *lapB *gene was represented by a pseudogene in *M*. *haemolytica *serotype 1 str. PHL101 as explained above. However, Caskey et al. [30] have shown that this strain is able to transport L-arginine with a high efficiency, something that seems unlikely if LapB constitutes an integral membrane protein subunit (ArtM or ArtQ) of an L-arginine ABC transporter as predicted by Highlander et al. [27]. These results support that the *lapB *operon is functionally unrelated to the other *lap *genes.

Two previous works have revealed that the genes encoding the different subunits of the L-arginine ABC transporter ArtQMP_2 _and the periplasmic binding proteins, ArtI and ArtJ, belong to the gene string *artP*-*artI*-*artQ*-*artM*-*artJ *in *E*. *coli *[29,48]. Although the genes code for functionally and physically interacting proteins, the results of Wissenbach et al. [48] suggest that they are organized into two separate operons: *artPIQM *and *artJ*. The results obtained from STRING searches highlighted two aspects (Figure 2). On one hand, the *artI *gene is present in six genomes, including those of *Pasteurellaceae*, where it belongs to the conserved gene string *artP*-*artI*-*artQ*-*artM*. On the other hand, the *artJ *gene is present in only the enterobacterial genomes of *E*. *coli *O157:O7 str. Sakai and *S*. Typhi strain Ty2 where it is connected to the conserved gene string *artP*-*artI*-*artQ*-*artM*. These results are compatible with our initial search for putative orthologs which retrieved two closely related proteins, ArtI and ArtJ, in a subset of the genomes, suggesting that the *artJ *gene is absent from most genomes. For each *art *gene we searched the genome of *M*. *haemolytica *serotype 1 str. PHL213 in order to identify putative orthologs. We found a main cluster of typical genes, *artP*-*artI*/*artJ*-*artQ*-*artM*, on contig22 and a single gene, *lapT*, on contig74 (data not shown). Highlander et al. [27] have shown that *lapT *is transcribed from a single promoter, suggesting that the gene comprises its own operon, while we have shown that the identified genes on contig22 are transcribed in the same direction (data not shown). These results are compatible with the predictions of Wissenbach and coworkers [29,48] and support that the *artPIQM *and *lapT *genes are organized into two separate operons, thus reaffirming our hypothesis that *lapT *and *artJ*, but not *artI*, are orthologs of each other.

The *xylA *and *xylB *genes were predicted to code for proteins that functionally interact in the dissimilation of D-xylose through the pentose phosphate pathway. The *xylA *and *xylB *genes from *E*. *coli *are organized into a single operon [49]. STRING searches revealed that the *xylA *and *xylB *genes are present in four genomes, including only one *Pasteurellaceae *genome, where they belong to the conserved gene string *xylA*-*xylB *(Figure 2). In accordance with these results, we have shown that the *xylA *and *xylB *genes are transcribed in the same direction (Figure 1), supporting that the respective genes comprise an operon.

## Discussion

Table 1 presents the *Mannheimia *genomes analyzed in this study, sorted by their overall similarity to *M*. *haemolytica *serotype 1 str. PHL213. The dataset includes the type strains of each species, except *M*.*ruminalis *where the type strain HPA92^T ^has lost the *lkt *operon [26], and represents the diversity within genus *Mannheimia *based on phenotypic characters (biogroups), 16S rRNA phylogeny, and geographic origin. Most *Mannheimia *species are inhabitants of the respiratory tract, but the genomes analyzed here encompass a wide variety of lifestyles, including potential pathogens of the ruminant respiratory tract (*M*. *haemolytica *+ *M*. *glucosida *and *M*. *varigena*), one commensal of the ovine rumen (*M*. *ruminalis*), and one potential pathogen of the ruminant integument (*M*. *granulomatis*).

Prior to this work, Larsen et al. [26] used a strategy that combines phylogenetic and compositional methods to explore the evolution of the *lkt *operon in genus *Mannheimia*. This analysis suggested that the operon has been vertically inherited from the last common ancestor of genus *Mannheimia *to any ancestor of the diverging subclades, although several strains belonging to *M*. *ruminalis *have lost the operon. However, reconstruction of the *rtxA *tree provided strong support for a sister group relationship between *M*. *haemolytica *+ *M*. *glucosida *and *M*. *ruminalis *on one hand and the more distantly related taxon [*Pasteurella*] *trehalosi *on the other. This incongruence between gene trees and organismal phylogenies could arise from HGT of the *lkt *operon from [*P*.] *trehalosi *to a common ancestor of these *Mannheimia *subclades.

In this article, we have examined the compatibility of the 5' flanking gene order with the Larsen and coworkers' [26] hypothesis of vertical inheritance. Given the collection of strains, information on the operon organization of the 5' flanking genes, and an organismal phylogeny that describes bifurcation order, the 5' flanking gene order of their ancestors can be inferred. The results of Highlander et al. [27] suggested that the gene cluster *lapCABT *codes for functionally and physically interacting proteins in an L-arginine ABC transporter from *M*. *haemolytica *serotype 1 str. PHL101. However, our results indicate that these genes comprise three functionally unrelated operons: *hslVU *(*lapCA*), *lapB*, and *artJ *(*lapT*). The most probable evolutionary scenario to explain the phylogenetic origin and distribution pattern of individual 5' flanking regions is shown in Figure 1. The 5' flanking gene strings, *hslVU*-*lapB*-*artJ*-*lktC *and *xylAB*-*lktC*, are peculiar to *M*. *haemolytica *+ *M*. *glucosida *and *M*. *granulomatis*, respectively, whereas the gene string *hslVU*-*lapB*-*lktC *is present in *M*. *ruminalis*, the supposed sister group of *M*. *haemolytica *+ *M*. *glucosida*, and in the most ancient subclade *M*. *varigena*. In *M*. *granulomatis*, we found remnants of the gene string *hslVU*-*lapB*-*lktC *in the *xylB*-*lktC *intergenic region as explained below. These observations indicate that the gene string *hslVU*-*lapB*-*lktC *is more ancient than the *hslVU*-*lapB*-*artJ*-*lktC *and *xylAB*-*lktC *gene strings. The disruption of the 5' flanking gene order in the genomes of *M*. *haemolytica *+ *M*. *glucosida *can be explained by two different plausible evolutionary scenarios. On one hand, the absence of the *artJ *operon from the closely related *Pasteurellaceae *genomes and the presence of the operon in the enterobacterial genomes of *E*. *coli *O157:O7 str. Sakai and *S*. Typhi strain Ty2 suggest that it has been acquired by HGT. On the other hand, the insertion of the *artJ *operon could also be due to translocation of a vertically inherited *artJ *operon and parallel loss of the operon in the closely related *Pasteurellaceae *genomes. In a recent work, Larsen et al. [26] used the average relative 3:1 dinucleotide abundance to identify horizontally transferred genes among a dataset of 56 annotated genes from *M*. *haemolytica *and noted that the *lapT *(*artJ*) gene was non-deviant from the average genome signature, thus opposing the hypothesis of HGT. It should be noted that false negatives (missed transferred genes) arise when genes have ameliorated due to the mutational processes affecting the recipient genome or the genes are closely related to the recipient genome in terms of context bias [50]. However, the young age of such a transfer (i.e. during early evolution of *M*. *haemolytica *+ *M*. *glucosida*) and the absence of the *artJ *operon from the closely related *Pasteurellaceae *genomes make these scenarios unlikely. Therefore, the disruption of the 5' flanking gene order in the genomes of *M*. *haemolytica *+ *M*. *glucosida *is compatible only with a history of translocation of the *artJ *operon during early evolution of this subclade (Figure 1). One of the unexpected findings of the first comparisons of the 5' flanking regions was the presence of a unique *xylAB *operon immediately upstream of the *lkt *operon in *M*. *granulomatis *which diverged after *M*. *varigena *but prior to *M*. *ruminalis *and *M*. *haemolytica *+ *M*. *glucosida*. We have found a weak but significant hit to the *hslVU *operon in the *xylB*-*lktC *intergenic region, reflecting the presence of highly fragmented remnants of the ancient gene string *hslVU*-*lapB*-*lktC*. The scenario derived from this analysis is compatible with a history of inactivation and multistep deletion of the *hslV*, *hslU*, and *lapB *genes after divergence from the remaining *Mannheimia *subclades (Figure 1).

The results of this analysis are compatible with the Larsen and coworkers' [26] hypothesis of vertical inheritance as reflected in Figure 1. Following this hypothesis, under the assumption that closely related species accumulate fewer rearrangements than the distant ones, it is expected that the *lkt *operons belong to gene strings that are conserved in all genomes, whereas HGT would result in disruption of the 5' flanking gene order (breakpoints) between the same genomes. Indeed, we found (remnants of) the ancient gene string *hslVU*-*lapB*-*lktC *among any subclades within genus *Mannheimia*, suggesting that it has been vertically inherited from the last common ancestor of genus *Mannheimia *to any ancestor of the diverging subclades. The presence of individual 5' flanking regions in *M*. *haemolytica *+ *M*. *glucosida *and *M*. *granulomatis *reflects later genome rearrangements within each subclade. Thus, the incongruence observed in the *rtxA *tree [26] points towards two most probable explanations. On one hand, it is compatible with a history of HGT from a common ancestor of *M*. *haemolytica *+ *M*. *glucosida *and *M*. *ruminalis *to [*P*.] *trehalosi*. On the other hand, it may reflect a history of homologous recombination between vertically inherited *lktA *genes.

While the set of *Mannheimia *genomes allowed us to study the phylogenetic origin of the *lkt *operon, we cannot rule out additional divergences within the 5' flanking region at or below the subclade level. For example, the analysis of the promoter activity of the 5' region in *M*. *haemolytica *serotype 1 str. PHL101 has shown that transcriptional coupling between the divergently arranged *artJ *and *lkt *promoters is important for coordinating high level expression of the *lkt *operon [51]. Thus, during early evolution of *M*. *haemolytica *+ *M*. *glucosida*, the acquisition of a preformed transcriptional enhancer has occurred which could explain the increased potential of certain *M*. *haemolytica *+ *M*. *glucosida *strains to cause a particular type of infection. However, in order to determine whether the evolution of novel 5' flanking regions has contributed to pathoadaptive niche differentiation within genus *Mannheimia*, we need to examine the distribution pattern of individual 5' flanking regions by including additional strains belonging to each subclade.

## Conclusion

To sum up, two conclusions have been outlined. On one hand, the presence of (remnants of) the ancient gene string *hslVU*-*lapB*-*lktC *among any subclades within genus *Mannheimia *supports that it has been vertically inherited from the last common ancestor of genus *Mannheimia *to any ancestor of the diverging subclades, thus reaffirming the Larsen and coworker's [26] hypothesis of vertical inheritance of the *lkt *operon On the other hand, acquisition of individual 5' flanking regions has occurred in *M*. *haemolytica *+ *M*. *glucosida *and *M*. *granulomatis*.

## Methods

### Taxa used

For this study we included *M*. *haemolytica *serotype 1 str. PHL213, *M*. *haemolytica *serotype 2 str. CCUG 12392^T^, *M*. *glucosida *serotype 11 str. P925^T^, *M*. *ruminalis *str. HPA113, *M*. *granulomatis *str. P1135/26^T^, and *M*. *varigena *str. 177^T ^to balance the number of representatives from the four major subclades within genus *Mannheimia *based on phenotypic characters (biogroups), 16S rRNA phylogeny, geographic origin, and lifestyle (see Table 1 for a description of strains used in this study, along with their accession numbers). All strains in our analysis have been represented in previous studies by Angen et al. [18,47,52,53] and Larsen et al. [26].

### DNA isolation, amplification, and sequencing

We retrieved data (contig74) from the annotated genome sequence of *M*. *haemolytica *serotype 1 str. PHL213. For the remaining *Mannheimia *strains, we constructed, screened, and sequenced phagemid libraries. We constructed a 542 bp probe in the *lktCA *region from *M*. *haemolytica *serotype 1 str. PHL213 by using the forward primer lktc_UP (5'-CCCTCCACAAAGAATGGAGCTG-3') in conjunction with the reverse primer lkta_DOWN (5'-TTTTGGTTGCCGTTAAAGTGTT-3'). The reaction conditions were 2.5 U *Taq *polymerase, 16 mM (NH_4_)_2_SO_4_, 67 mM Tris-HCl, 0.01% Tween-20, 2.5 mM Mg_2_SO_4_, each primer at 0.5 mM, and each nucleotide at 0.1 mM. The cycling conditions were initial denaturation at 94°C followed by 25 cycles of 94°C for 30 s, 52°C for 30 s, and 72°C for 30 s, finishing with extension at 72°C for 10 min. The PCR product was labeled with digoxigenin-11-dUTP by using the Random Primed DNA Labeling Kit according to the manufacturer's instructions (Roche). To construct phagemid libraries, we partially digested genomic DNA with the restriction enzyme *Sau*3AI. Aliquots were run on agarose gels and fragments of approximately 3–6 kb were cloned into the *Bam*HI site of the Zap Express vector according to the manufacturer's instructions (Stratagene). The ligation mixture was packaged *in vitro *into the Gigapack III Gold Packaging Extract and transfected into *E*. *coli *XL1-Blue MRF' according to the manufacturer's instructions (Stratagene). The plaques were lifted and crosslinked to a 0.45 μm nitrocellulose membrane (Millipore) according to the manufacturer's instructions (Stratagene). The phagemid libraries were screened for *lktCA *clones and the pBK-CMV phagemid vector was *in vivo *excised from the ZAP Express vector by using the ExAssist helper phage and *E*. *coli *XLOLR according to the manufacturer's instructions (Stratagene). The plasmids were then directly sequenced.

### Analysis of genes, pseudogenes, and remnant DNA

ORFs were considered to be genes if they showed a significant database hit, or were longer than 100 amino acids, or were longer than 50 amino acids and conserved between at least two genomes in the data set. Sequences with database matches to a functional gene but spanning more than one ORF were considered to be pseudogenes. Significant sequence similarities between unique genes or gene strings in one genome and intergenic regions located at the corresponding sites in other genomes were considered to be remnant DNA.

For each ORF we searched the NCBI Genome database by using PSI-BLAST [54] with default parameters until convergence in order to identify putative orthologs. BLAST searches were performed with a very stringent cut-off value (E = 1E-50) to minimize problems associated to BLAST identifications. The annotation of each sequence and the corresponding multiple alignments were revised individually to discard wrongly identified putative orthologs. Functional characterization of genes was assessed by searching the SWISS-PROT database [55], the Clusters of Orthologous Groups of proteins (COG) database [56], and the Protein Data Bank (PDB) by using PSI-BLAST [54] with default parameters until convergence.

Conservation of gene order is one of the principal types of genome context information and is considered to be an indication of operon organization of the respective genes and a legitimate predictor of functional and potentially physical interactions between genes [4]. We selected 17 proteobacterial genomes for analysis of gene order conservation (Table 3). The data set included a balanced number of representatives from the gamma-proteobacterial group, including three *Pasteurellaceae *genomes which are closely related to the *Mannheimia *genomes, and the beta-proteobacterial sister group. For each protein we performed searches at the STRING server [57] with a high confidence score > 0.7.

## Authors' contributions

JL carried out the molecular genetic studies, participated in the sequence annotations and in the interpretation of the results, and drafted the manuscript. PK and JF participated in the design of the study. HC participated in the analysis of gene order data. MB was responsible for the primary strain collection and participated in the interpretation of the results. JEO conceived of the study, participated in its design and coordination, and helped to draft the manuscript. All authors read and approved the final manuscript.
